# The Influence of Panoramic Radiograph Quality on the Accuracy of AI-Based Tooth Detection

**DOI:** 10.3390/diagnostics16111650

**Published:** 2026-05-27

**Authors:** Julien Issa, Reinier Hoogeveen, Marta Dyszkiewicz-Konwińska, Erwin Berkhout

**Affiliations:** 1 Department of Oral Radiology & Digital Dentistry, Academic Center for Dentistry Amsterdam (ACTA), University of Amsterdam & Vrije Universiteit Amsterdam, 1081 LA Amsterdam, The Netherlands; 2 ACTA|AI Lab, Academic Center for Dentistry Amsterdam (ACTA), University of Amsterdam & Vrije Universiteit Amsterdam, 1081 LA Amsterdam, The Netherlands; 3 Department of Diagnostics, Poznan University of Medical Sciences, 60-812 Poznan, Poland

**Keywords:** artificial intelligence, dental imaging, deep learning, clinical decision support

## Abstract

**Objectives**: This study aimed to evaluate the influence of panoramic radiograph quality on the performance of an artificial intelligence (AI)-based tooth detection system and to identify specific image quality criteria associated with detection accuracy. **Methods**: A total of 424 panoramic radiographs were retrospectively selected from a clinical database. Radiographic quality was assessed using a modified Clinical Image Evaluation Chart, including criteria related to bite block presence, anteroposterior positioning, occlusal plane curvature, patient movement, anatomical coverage, overlapping contact points, air gap, contrast, cervical spine overlap, symmetry of the ascending mandibular ramus, and the number of visible teeth. Automated tooth detection was performed using a convolutional neural network based on the Mask R-CNN architecture (SynbrAIn, Italy). AI detection outputs were validated against expert human evaluation. Spearman’s rank correlation analyses were conducted to assess associations between individual image quality criteria and the number of AI detection errors per radiograph. **Results**: Significant negative associations were observed between AI detection errors and the number of visible teeth (ρ = −0.311, *p* < 0.001), presence of a bite block (ρ = −0.248, *p* < 0.001), reduced patient movement (ρ = −0.204, *p* < 0.001), correct anteroposterior positioning (ρ = −0.165, *p* < 0.001), and overall image quality score (ρ = −0.120, *p* = 0.010). In contrast, the presence of an air gap above the anterior teeth (ρ = 0.099, *p* = 0.042) and overlapping contact points (ρ = 0.122, *p* = 0.012) were positively associated with increased detection errors. No significant associations were identified for occlusal plane curvature, contrast, cervical spine overlap, anatomical coverage, or mandibular ramus symmetry. Overall, the AI system was more sensitive to indicators of anatomical completeness and patient positioning than to minor radiographic imperfections. **Conclusions**: Panoramic radiograph quality, particularly indicators of anatomical completeness and patient positioning, is associated with the performance of AI-based tooth detection. While the AI system demonstrated robustness to common image quality variations, adherence to standardized acquisition protocols remains important to minimize detection errors.

## 1. Introduction

Panoramic radiographs are commonly used in dentistry as an imaging modality for both diagnostic and treatment planning [1]. Its broad field of view allows for the simultaneous visualization of dentition, alveolar bone, temporomandibular joints, maxillofacial structures, and adjacent anatomical regions [1]. Despite these advantages, panoramic radiographs inherently represent a two-dimensional projection of complex three-dimensional anatomy, which introduces notable limitations [2,3,4]. These include geometric distortion, non-uniform magnification, and superimposition of anatomical structures, all of which may obscure critical diagnostic information [2,3,4].

In addition to modality-related constraints, diagnostic interpretation of panoramic radiographs is influenced by clinician-dependent factors, including level of experience, perceptual bias, cognitive fatigue, and time pressure [5,6]. These factors contribute to substantial inter- and intra-observer variability [6]. Collectively, these challenges underscore the need for objective, standardized, and reproducible diagnostic support tools that can reduce interpretative subjectivity and enhance consistency in radiographic evaluation. The development and integration of such tools are increasingly viewed as essential for improving diagnostic reliability in routine clinical workflows.

In recent years, this variability and subjectivity in radiographic interpretation have accelerated the development of artificial intelligence (AI) applications in dental imaging [7]. AI has emerged as a transformative technology capable of automating image analysis tasks, including object detection, classification, and segmentation, thereby supporting clinicians in diagnostic decision-making [7]. AI-based systems have demonstrated the ability to identify minute pixel-level variations that may escape human detection, highlighting their potential for enhanced diagnostic precision [8]. Convolutional neural networks (CNNs), a subset of deep learning architectures, demonstrated strong performance across a range of clinically relevant tasks, including tooth detection and numbering, caries identification, periodontal bone loss measurement, periapical lesion detection, and anatomical structure segmentation [9,10,11,12]. These results collectively suggest that AI can enhance diagnostic reliability, reduce observer bias, and potentially streamline radiographic workflows.

Despite these advancements, AI models remain susceptible to errors, and diagnostic accuracy remains inherently dependent on the quality of the input data. Panoramic radiographs acquired in routine clinical practice often exhibit considerable variability due to patient positioning errors, motion artifacts, improper exposure settings, and device-specific limitations. In a large-scale evaluation of panoramic radiographs, Dhillon et al. [13] reported that only 11% of images were free from positioning errors, while 89% exhibited at least one form of inaccuracy, underscoring the prevalence of suboptimal image quality in everyday practice.

While the impact of image quality on human diagnostic performance has been extensively studied, its influence on AI-based segmentation and detection systems remains insufficiently explored. Most existing AI studies have relied on curated datasets composed of high-quality images acquired under controlled conditions, which may not accurately reflect real-world clinical variability [14]. Consequently, the generalizability and robustness of AI systems when confronted with variations in sharpness, contrast, and anatomical coverage remain uncertain. AI models that perform well under ideal conditions may degrade when applied to images of lower or inconsistent quality, potentially compromising diagnostic reliability.

The aim of this study is to evaluate the impact of panoramic radiograph quality on the performance of an AI-based tooth segmentation system. Specifically, this investigation assesses how variations in image sharpness, contrast, and anatomical coverage influence the accuracy of automated tooth identification.

## 2. Materials and Methods

### 2.1. Research Design and Dataset

This retrospective, double-blind, randomized cohort study was approved by the Ethics Review Committee of the Academic Centre for Dentistry Amsterdam (ACTA; protocol number 2025-21887). This study was designed and reported in accordance with the STARD-AI guidelines for diagnostic accuracy studies involving artificial intelligence.

A total of 500 panoramic radiographs were retrospectively retrieved from the database of Amphia Hospital (Tilburg, the Netherlands). All images were anonymized and pre-randomized based on their unique SOP Instance Numbers prior to selection. The panoramic radiographs were acquired using a single panoramic unit (Orthophos XG 5, Dentsply Sirona, Bensheim, Germany) and included both large- and small-field exposures. The study population comprised patients aged 10 to 95 years. The sample size was chosen to ensure adequate representativeness while maintaining feasibility for detailed manual image quality assessment.

**Figure 1:**
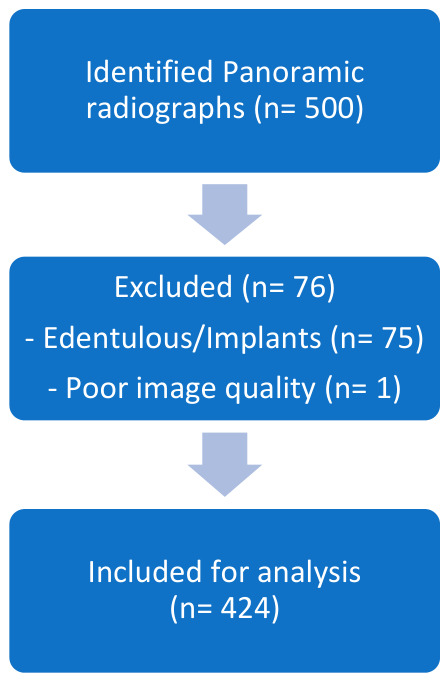
Flowchart illustrating the radiograph selection and exclusion process.

Prior to analysis, seventy-six radiographs were excluded. Exclusion criteria comprised panoramic radiographs of fully edentulous patients or cases containing only dental implants, as these conditions preclude meaningful evaluation of natural tooth segmentation. Additionally, radiographs with severely compromised image quality that prevented reliable identification of dental structures by both the AI system and human assessors were excluded. After applying these criteria, 424 panoramic radiographs were included in the final analysis (Figure 1).

### 2.2. Assessment of the Radiograph Quality

All panoramic radiographs were independently evaluated by five experts: four oral and maxillofacial radiologists with more than ten years of clinical experience and one final-year dental student. Image quality was assessed using a modified version of the Clinical Image Evaluation Chart described by Choi et al. [4] (Table 1).

The modified assessment tool incorporated both technical and anatomical criteria, including the presence of a bite block, patient positioning errors, artifacts, and ghost images, as well as anatomical coverage and radiographic clarity parameters such as sharpness, brightness, and contrast. The number of visible teeth was also recorded; however, this variable was interpreted as patient- or anatomical-completeness factor rather than a technical image-quality factor. Each criterion was assigned an ordinal score reflecting its presence, absence, or degree of adequacy, yielding a maximum cumulative score of 68 points per radiograph. Higher scores indicated superior diagnostic quality. Features such as complete visualization of the condyles, correct anteroposterior positioning, absence of motion artifacts, and appropriate brightness and contrast contributed positively to the overall score, whereas deficiencies, including overlapping teeth, ghost images, and motion artifacts, reduced the score. The inclusion of multiple sub-criteria (e.g., mandibular ramus symmetry, cervical spine visibility, and patient movement) enabled a comprehensive assessment of both image acquisition quality and diagnostic interpretability.

The individual image-quality scoring datasets generated by each assessor were subsequently merged into a single dataset for analysis. For each image quality criterion, the ordinal scoring system described in Table 1 was recorded, and consensus scores were determined by the mode when discrepancies occurred between assessors. The mode was used because the image quality variables were ordinal and categorical in nature, thereby preserving the most frequently assigned observer score while avoiding artificial averaging of ordinal categories. In cases where no single mode was present, discrepancies were reviewed and resolved by consensus discussion. This approach ensured consistency with the original scoring framework and facilitated the construction of a statistically robust dataset for subsequent analyses. Following image quality assessment, each radiograph was annotated by identifying present teeth and assigning tooth numbers according to the FDI tooth numbering system.

Prior to scoring and annotation, all observers participated in a calibration session using a standardized PowerPoint presentation containing representative panoramic images with predefined scores for each criterion. This calibration aimed to harmonize interpretation and reduce inter-rater variability. To assess inter-rater reliability, each assessor independently evaluated a subset of 25 radiographs (approximately 5% of the dataset, comprising 119 unique scoring instances). Agreement among raters was quantified using the intraclass correlation coefficient (ICC).

### 2.3. AI Model and Automated Evaluation

**Figure 2:**
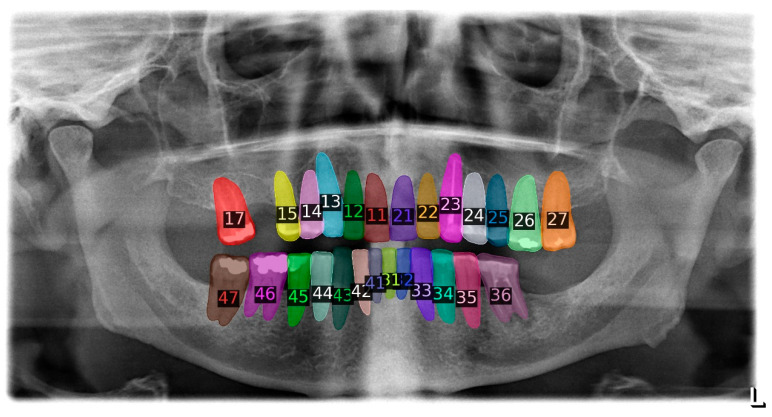
Panoramic radiograph analyzed by the AI-based tooth detection system.

Automated tooth detection was performed using SynbrAIn (SynbrAIn S.r.l., Milan, Italy), a pre-trained artificial intelligence system for tooth detection and numbering on panoramic radiographs [15]. The model is based on a Mask Region-based Convolutional Neural Network (Mask R-CNN), an instance segmentation architecture that enables object detection, tooth classification, and pixel-level segmentation [15]. The model used in the present study was not trained or fine-tuned on the radiographs included in this dataset. The model was applied to the panoramic radiographs to automatically detect and label individual teeth (Figure 2). For each radiograph, the AI-generated output was exported to a spreadsheet format, where the presence or absence of each tooth position was recorded as a binary variable (1 = tooth detected, 0 = tooth not detected).

### 2.4. Statistical Analysis

To evaluate the association between panoramic radiograph quality and AI-based analysis performance, Spearman’s rank correlation coefficient was calculated between the total image quality score and the number of AI segmentation errors. This non-parametric test was selected because the segmentation error counts exhibited a non-normal distribution, as confirmed by visual inspection of histograms (Appendix A).

In addition, separate Spearman correlation analyses were performed for each image quality sub-criterion to identify specific technical or anatomical factors most strongly associated with AI segmentation errors. All statistical analyses were conducted using IBM SPSS Statistics (version 26). Statistical significance was set at *p* < 0.05, and two-tailed tests were applied to evaluate correlations in both positive and negative directions.

## 3. Results

### 3.1. Quality of the Panoramic Radiograph

Across the analyzed dataset, image quality scores ranged from 28 to 68, with a mean score of 52.07 ± 6.15. The maximum attainable score of 68 was observed in seven radiographs. The most frequently observed quality deficiency was the presence of an air gap above the maxillary anterior roots, which occurred in 63% of images. Patient movement was the least frequently observed defect, present in 1% of radiographs.

### 3.2. AI-Based Tooth Detection

**Figure 3:**
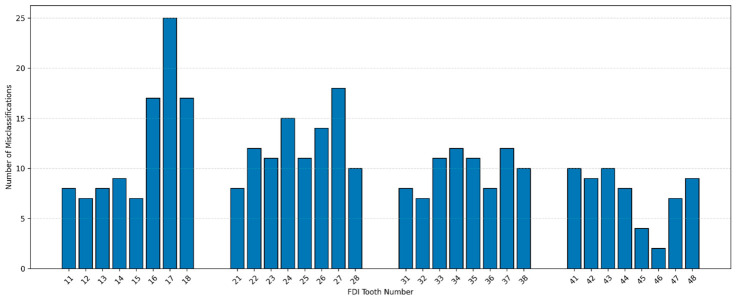
Frequency of AI-based tooth detection errors per tooth according to the FDI numbering system.

In total, at least one AI-based detection error occurred in 117 of the 424 panoramic radiographs (28%). The number of detection errors per radiograph ranged from 0 to 24, with a mean of 0.7 errors per image. Error frequency varied across tooth positions, with higher counts observed in posterior regions compared with anterior teeth. The highest number of errors was recorded for tooth 17 (*n* = 25), whereas the first anterior tooth to appear in the ranking was tooth 22 (*n* = 12), which ranked ninth overall. Fewer detection errors were observed in the mandible (*n* = 130) than in the maxilla (*n* = 190). The distribution of AI detection errors per tooth, according to the FDI tooth numbering system, is shown in Figure 3.

### 3.3. Inter-Rater Reliability

Inter-rater reliability among the four co-assessors demonstrated good agreement, with an intraclass correlation coefficient (ICC) of 0.841.

### 3.4. Association Between Overall Image Quality and AI-Based Detection Errors

**Figure 4:**
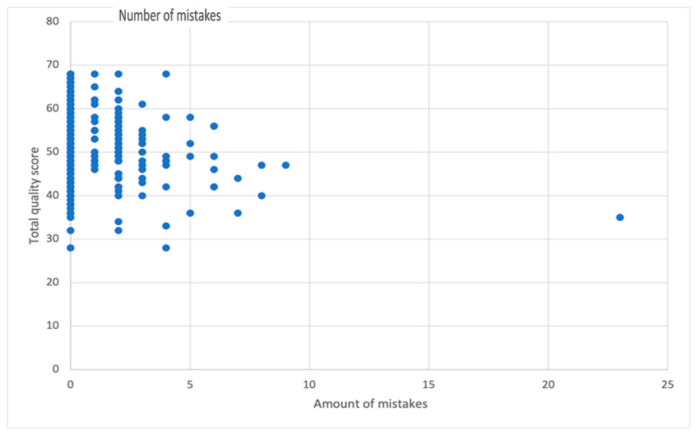
Scatterplot illustrates the relationship between total image quality score and the number of AI-based detection errors.

Spearman’s rank correlation analysis demonstrated a weak but statistically significant negative association between the total panoramic radiograph quality score and the number of AI detection errors per image (ρ = −0.120–0.120, *p* = 0.014; Table 2). Because higher chart scores represent better acquisition quality, this finding indicates that lower-quality panoramic radiographs were associated with slightly higher numbers of AI detection errors. This relationship is illustrated in Figure 4.

### 3.5. Association Between Individual Image Quality Factors and AI-Based Detection Errors

Analysis of individual image quality-related criteria identified several statistically significant associations with AI detection errors (Table 2). The presence of a bite block was associated with fewer AI-based detection errors (ρ = −0.248, *p* < 0.001), and patient movement showed a similar association (ρ = −0.204, *p* < 0.001). Correct anteroposterior positioning was also significantly associated with lower error counts (ρ = −0.165, *p* = 0.001).

In contrast, the presence of an air gap above the anterior teeth (ρ = 0.099, *p* = 0.042) and overlapping contact points between adjacent teeth (ρ = 0.122, *p* = 0.012) were associated with higher numbers of AI-based detection errors. Overall, these findings indicate that image-quality deficiencies were consistently associated with increased AI error counts. No statistically significant associations were observed for artifacts, ghost images, condylar visibility, contrast, cervical spine overlap, or periodontal ligament visibility.

### 3.6. Association Between the Number of Visible Teeth and AI-Based Detection Errors

A statistically significant negative correlation was observed between the total number of visible teeth and the number of AI detection errors per panoramic radiograph (ρ = −0.311, *p* < 0.001; Table 2). Similar associations were observed for the number of maxillary teeth (ρ = −0.210, *p* < 0.001) and mandibular teeth (ρ = −0.175, *p* < 0.001).

### 3.7. Association Between the Occlusal Plane and AI-Based Detection Errors

No statistically significant association was found between occlusal plane curvature and the number of AI detection errors per panoramic radiograph (ρ = −0.012, *p* = 0.812; Table 2).

## 4. Discussion

This study evaluated the extent to which panoramic radiograph quality is associated with the performance of an AI-based tooth detection system under routine clinical conditions. A weak but statistically significant inverse association was observed between overall image quality and the number of AI detection errors (ρ = −0.120, *p* = 0.014), indicating that higher-quality panoramic radiographs were associated with slightly improved AI performance. However, the very small magnitude of this association suggests that overall image quality explained only a limited proportion of the variation in AI-based detection errors. This may indicate that the AI system was relatively robust to routine image-quality variations, possibly because tooth detection and numbering rely mainly on the visibility, morphology, and spatial arrangement of teeth rather than on all aspects of general diagnostic image quality.

The observed association between overall image quality and AI performance is consistent with previous findings demonstrating improved diagnostic accuracy when AI models are trained or evaluated on high-quality panoramic radiographs [16]. These findings underscore the need for standardized radiographic acquisition protocols, especially when AI is integrated into dental diagnostics, where image quality may vary considerably [13]. However, unlike many prior studies that excluded radiographs with positioning errors or reduced image quality, which potentially contribute to overly optimistic estimates of AI performance [17,18], the present study intentionally included images reflecting real-world clinical variability. This approach enhances external validity and supports the clinical relevance of the findings, particularly in settings where optimal acquisition conditions cannot always be ensured.

Among individual image quality-related criteria, patient positioning emerged as a key determinant of AI performance. Correct anteroposterior positioning and the use of a bite block were both significantly associated with fewer detection errors. The presence of a bite block (ρ = –0.248, *p* < 0.001) demonstrated the strongest association, underscoring its role in stabilizing patient position and maintaining appropriate spatial relationships between anatomical structures. These findings align with earlier reports indicating that omission of a bite block substantially increases positioning errors in panoramic imaging [19]. Patient movement was also associated with AI-based detection errors (ρ = −0.204, *p* < 0.001); however, given the low prevalence of motion artifacts in the dataset (5 radiographs), this finding should be interpreted cautiously. This minimal variation could limit generalizability and inflate the statistical association [20]. To better quantify the influence of motion, future studies should include more deliberately selected or simulated low-quality images. In contrast, occlusal plane curvature did not show a significant association with AI-based detection errors. This suggests that, within the range of variations observed in routine clinical imaging, occlusal plane deviations may exert a limited influence on AI performance. Notably, this contrasts with the initial hypothesis and highlights that not all visually apparent positioning deviations necessarily impair AI-based analysis. One possible explanation is that Mask R-CNN-based models extract hierarchical image features at multiple spatial levels, allowing the model to identify tooth-like structures despite moderate geometric variation in the dental arch. As long as individual teeth remain sufficiently visible and retain recognizable morphology and relative spatial relationships, variations in occlusal plane curvature may not substantially affect detection or numbering performance.

Certain quality deficiencies, including the presence of an air gap above the anterior maxillary teeth (ρ = 0.099, *p* = 0.042) and overlapping contact points (ρ = 0.122, *p* = 0.012), were positively associated with detection errors. Although these correlations were weak, their clinical relevance should not be underestimated. The air gap was the most frequently observed positioning error in the dataset, affecting nearly two-thirds of the radiographs, consistent with prior literature identifying improper tongue positioning as a common acquisition error [13]. Even subtle disruptions in anterior anatomical visibility may therefore introduce localized uncertainty that affects AI predictions.

The robustness of the AI system despite these localized deficiencies may be partly explained by the availability of broader anatomical context [21]. This interpretation is supported by the strong inverse association observed between the number of visible teeth and AI detection errors. Radiographs with a higher number of present teeth, both overall and within each jaw, were associated with improved AI performance, suggesting that a richer anatomical context may help compensate for local image distortions. Similar observations have been reported by Ali et al. [22]; an increase in AI error rates was reported in regions adjacent to missing posterior teeth, where anatomical landmarks are reduced. These increased AI errors may be explained not only by reduced anatomical context but also by tooth-position changes secondary to tooth loss. Missing teeth can result in drifting, migration, or mesial/distal drift of adjacent teeth, thereby altering the expected dental arch configuration and spatial relationships on which AI models may rely for tooth localization and numbering.

Several commonly researched image quality-related factors, including artifacts, ghost images, cervical spine overlap, condylar visibility, contrast, and periodontal ligament visibility, were not significantly associated with AI detection errors. This finding aligns with the previous work of Tuzoff et al. [11], indicating that CNNs may tolerate moderate image imperfections without substantial performance degradation. From a clinical perspective, this is reassuring, as it suggests that minor technical deficiencies may not preclude effective AI-supported radiographic analysis.

Several limitations should be acknowledged. First, the correlational design does not permit causal inference. In addition, the use of mode-based consensus scoring may have reduced the visibility of observer-level variability across individual image quality sub-criteria. Future studies should include sub-criterion-level agreement analyses to identify which image quality factors are most susceptible to observer variability. Second, although inter-rater reliability was high, image quality scoring and reference annotation remain subject to human judgment. Third, the evaluation was limited to a single AI system, and performance may differ across architectures or training strategies. Fourth, all panoramic radiographs were acquired using a single panoramic device. Although this reduced technical variability within the dataset, it may limit the generalizability of the findings to images acquired using other panoramic devices or imaging protocols. Device-related differences in image processing, exposure parameters, contrast, sharpness, magnification, and geometric distortion may influence both radiographic quality scores and AI-based tooth detection performance. Finally, the study focused on detection-level performance rather than pixel-wise segmentation accuracy.

From a clinical perspective, these findings indicate that panoramic radiograph quality and specific positioning-related criteria are associated with AI-based tooth detection performance, with generally small effect sizes. The AI system’s robustness to common variations in image quality suggests that AI-supported analysis may be feasible in routine clinical conditions. However, optimal patient positioning and adequate anatomical completeness remain clinically relevant for minimizing detection errors, particularly in complex cases. These results support integrating AI as a decision-support tool in dental practice, while emphasizing the importance of adhering to standardized acquisition protocols. Future research should validate these observations in larger and more diverse populations and investigate training strategies that explicitly incorporate real-world imaging variability to further enhance clinical reliability and generalizability.

## 5. Conclusions

This study demonstrates that several radiographic quality criteria, most notably the presence of a bite block, patient movement, anteroposterior positioning, air gap presence, contact point overlap, and the number of visible teeth, are associated with the accuracy of AI-based tooth detection. Although the association between overall panoramic radiograph quality and the number of AI detection errors was weak, it remained statistically significant, and the AI system showed considerable robustness to routine variations in image quality. These findings highlight that, while AI performance is not solely dependent on optimal imaging conditions, adherence to standardized, high-quality acquisition protocols remains important to support reliable clinical deployment of AI-based diagnostic tools.

## Figures and Tables

**Table 1:** Clinical image quality evaluation chart. * The number of visible teeth was recorded as patient/anatomical completeness.

Criteria	Sub-Criteria	Score
Bite Block	Present/Absent	4/0
Artifacts	Absent/Present, no overlap with alveolar process or elements/Overlap with alveolar process or elements	4/2/0
Ghost Images-Jewelry	Absent/Present, no overlap with alveolar process or elements/Overlap with alveolar process or elements	4/2/0
Ghost Images-Anatomy	Ghost image of anatomy not or barely visible/Ghost image of anatomy clearly visible	4/2
Anatomical Coverage	Both condyles visible/One condyle not fully visible/Both condyles not fully visible	4/2/0
	Inferior border of the mandible and inferior border of the orbit included/Inferior border of mandible or orbit not included	4/0
Patient Positioning	Occlusal plane: Slight curve/Flat/Strong V- or U-shape/Inverted curve	4/3/2/0
	Anteroposterior positioning: Correct/Anterior teeth narrowed or widened/Anterior teeth unclear or blurry	4/2/0
	Symmetry of ascending mandibular ramus: Equal width bilaterally/Asymmetry between left and right	4/0
	Patient movement: No interruptions/Cortical border of mandible exhibits step-like appearances	4/0
	Air gap above roots: No air gap/Air gap limits root visibility	4/0
	Overlapping teeth: No overlap/Overlap in <4 contact points/Overlap in >4 contact points	4/2/0
	Cervical spine influence: No influence on anterior teeth visibility/Cervical spine limits anterior teeth visibility	4/0
Sharpness, Brightness, and Contrast	Periodontal ligament space: Clearly visible/Poorly or not visible in <4 teeth/Poorly or not visible in >4 teeth	4/2/0
	Normal brightness and contrast/High contrast (“black-and-white” appearance)	4/2
Number of Teeth *	Maxilla: >10/6–9/<5	4/2/0
	Mandible: >10/6–9/<5	4/2/0
Total score		68

**Table 2:** Spearman’s correlation between individual image quality criteria and the number of AI-based tooth detection errors per panoramic radiograph. * *p* < 0.01.

Quality Factor	Mean ± SD	Correlation Coefficient (ρ)	*p*-Value
Overall quality	52.07 ± 6.15	−0.120	0.014 *
Bite block	2.7 ± 1.9	−0.248	*p* < 0.001 *
Artifacts	3.9 ± 0.6	0.043	0.377
Ghost images-jewelry	3.9 ± 0.5	0.042	0.385
Ghost images-anatomy	2.8 ± 1.0	−0.032	0.516
Inferior border orbit + mandible	3.6 ± 1.1	−0.084	0.085
Both condyles visible	3.1 ± 1.7	−0.021	0.662
Contrast	3.9 ± 0.3	−0.087	0.075
Anteroposterior positioning	3.2 ± 1.5	−0.165	0.001 *
Symmetry of ascending mandibular ramus	2.9 ± 1.8	0.049	0.314
Patient movement	4.0 ± 0.4	−0.204	*p* < 0.001 *
Air gap	1.5 ± 1.9	0.099	0.042 *
Overlap of contact points	1.6 ± 1.5	0.122	0.012 *
Cervical spine overlap	3.5 ± 1.3	−0.060	0.217
Periodontal ligament visibility	1.3 ± 1.6	−0.050	0.306
Number of teeth	25.65 ± 7.23	−0.311	*p* < 0.001 *
Number of teeth in maxilla	3.5 ± 1.1	−0.210	*p* < 0.001 *
Number of teeth in mandible	3.8 ± 0.7	−0.175	*p* < 0.001 *
Occlusal plane	3.0 ± 1.3	−0.012	0.812

**Figure A1:**
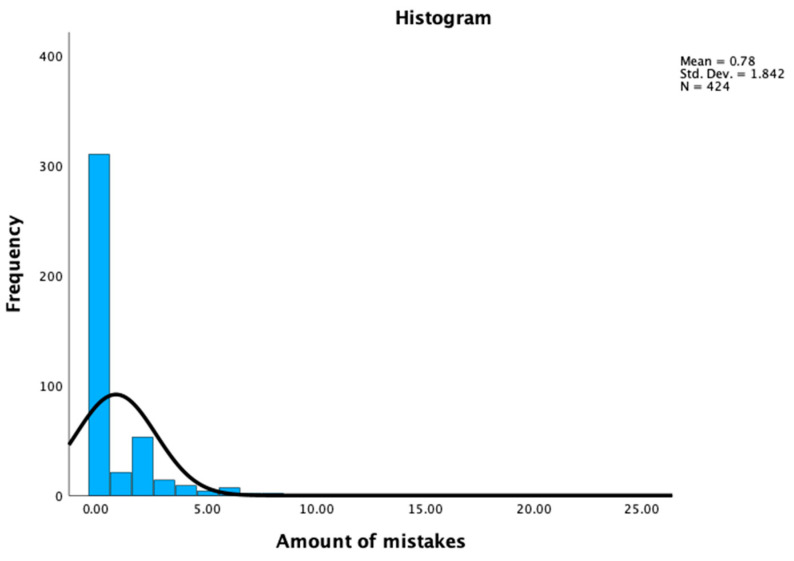
Frequency distribution of AI-based segmentation error rates.

## Data Availability

The data presented in this study are available on request from the corresponding author due to legal reasons.
